# Draft Genome Sequence of a Multidrug-Resistant New Delhi Metallo-β-Lactamase-1 (NDM-1)-Producing *Escherichia coli* Isolate Obtained in Singapore

**DOI:** 10.1128/genomeA.01020-13

**Published:** 2013-12-19

**Authors:** Eryu Xia, Wei Xin Khong, Kalisvar Marimuthu, Wenting Xu, Rick Twee-Hee Ong, Eng Lee Tan, Prabha Unny Krishnan, Brenda Sze Peng Ang, David Chien Lye, Angela Li Ping Chow, Yik-Ying Teo, Oon Tek Ng

**Affiliations:** NUS Graduate School for Integrative Science and Engineering, National University of Singapore, Singaporea; Institute of Infectious Disease and Epidemiology, Communicable Disease Centre, Tan Tock Seng Hospital, Singaporeb; Saw Swee Hock School of Public Health, National University of Singapore, Singaporec; Centre for Biomedical and Life Sciences, Singapore Polytechnic, Singapored; Department of Paediatrics, University Children’s Medical Institute, National University of Singapore, Singaporee; Department of Laboratory Medicine, Tan Tock Seng Hospital, Singaporef; Yong Loo Lin School of Medicine, National University of Singapore, Singaporeg; Life Sciences Institute, National University of Singapore, Singaporeh; Department of Statistics and Applied Probability, National University of Singapore, Singaporei; Genome Institute of Singapore, Agency for Science, Technology and Research, Singaporej

## Abstract

We report the draft genome sequence of a New Delhi metallo-β-lactamase-1 (NDM-1)-positive *Escherichia coli* isolate obtained from a surgical patient. The assembled data indicate the presence of 3 multidrug resistance plasmids, 1 of which shares 100% identity with an NDM-1 plasmid isolated previously from a nearby hospital, suggesting possible local transmission.

## GENOME ANNOUNCEMENT

Antimicrobial resistance in bacteria is a growing global public health problem. In its latest report, the United States CDC estimated that at least 2 million people became infected with antimicrobial-resistant bacteria, and >23,000 people die each year as a result (1). This rapid increase in antimicrobial resistance, coupled with the dwindling pipeline of effective antimicrobial armamentarium, has raised tremendous concern about the emergence of a doomsday organism. This concern is certainly warranted for the NDM-1-positive *Enterobacteriacae*, as bacteria that produce NDM-1 β-lactamase are typically resistant to a wide range of antibiotics, including carbapenems, one of the last-resort drugs for many bacterial infections (2, 3).

A 29-year-old male subject was admitted to Tan Tock Seng Hospital in 2010 for an accidental fall resulting in a 5th cervical spine fracture and spinal cord injury. He underwent surgical decompression, reconstruction, and stabilization with a good neurological outcome. The subject was in a rehabilitation ward with another patient. When this patient was identified as having an NDM-1 *Escherichia coli* infection, the subject had a rectal swab done as part of contact screening, as per the hospital protocol. Whole-genome sequencing (WGS) was performed on the NDM-1 *E. coli* isolate. Phenotypic characterization of the isolate identified resistance to at least 17 clinically used antibiotics, including second- and third-generation cephalosporins and carbapenems, but it remained susceptible to gentamicin and polymyxin B.

Genomic DNA was extracted from an overnight culture using MagNA Pure Compact (Roche Applied Science, Germany), and the Illumina Nextera XT sample prep kit was used to prepare the sequencing library for WGS on the Illumina MiSeq platform, generating 848,394 pass-filtered 250-bp paired-end reads. *De novo* assembly was performed using Velvet, with a *k*-mer value of 63, producing 242 contigs with a total length of 5,258,026 bp and an N_50_ of 90,507 bp. The contigs were then searched against the NCBI NT database (http://www.ncbi.nlm.nih.gov/genbank/index.html) using BLAST (4), identifying strain NA114 (accession no. CP002797.2) as the most probable *E. coli* strain, with a genomic coverage of 88.7%. Additionally, three circular plasmids were identified, with minimum 90% genomic coverage. Strains PECOS88 (accession no. CU928146.1) and pG-09EL71 (accession no. CP003303.1) were previously identified as *E. coli*. Interestingly, the third plasmid shares complete 100% identity with the NDM-1-encoding plasmid pTR3 (accession no. JQ349086.2), which was reported to be isolated from a patient admitted into another hospital in Singapore (5). This finding suggests possible epidemiological links between the two plasmids.

Resistance Gene Identifier (Institute of Infectious Disease Research, McMaster University [http://arpcard.mcmaster.ca]) was used to identify antibiotic resistance genes present in the isolate that showed complete concordance with the phenotypic results achieved by predicting resistance to β-lactams, tetracycline, rifampin, macrolides, sulfonamides, and fluoroquinolones (6). This demonstrated the utility of WGS for the identification of species and resistance to antibiotics with a single and more efficient workflow in clinical microbiology settings.

Further studies on the NDM-1-producing *E. coli* and other nosocomial multidrug-resistant bacterial isolates obtained from patients in Singapore are currently under way. These data may shed important light on the molecular mechanisms contributing to bacterial drug resistance dissemination and help guide preventive measures for hospital transmission.

### Nucleotide sequence accession numbers.

This whole-genome shotgun project has been deposited in EMBL under the accession no. CBVN010000001 to CBVN010000242.

## References

[1] Centers for Disease Control and Prevention (CDC) 2013 Antimicrobial resistance: threat report 2013. Centers for Disease Control and Prevention, Atlanta, GA. http://www.cdc.gov/drugresistance/threat-report-2013

[2] Nordmann P Poirel L Walsh TR Livermore DM 2011 The emerging NDM carbapenemases. Trends Microbiol. 19:588–595 22078325 10.1016/j.tim.2011.09.005

[3] Nordmann P Naas T Poirel L 2011 Global spread of carbapenemase-producing *Enterobacteriaceae*. Emerg. Infect. Dis. 17:1791–1798 22000347 10.3201/eid1710.110655 PMC3310682

[4] Camacho C Coulouris G Avagyan V Ma N Papadopoulos J Bealer K Madden TL 2009 BLAST+: architecture and applications. BMC Bioinformatics 10:421. 10.1186/1471-2105-10-421 20003500 PMC2803857

[5] Chen YT Lin AC Siu LK Koh TH 2012 Sequence of closely related plasmids encoding *bla*_NDM-1_ in two unrelated *Klebsiella pneumoniae* isolates in Singapore. PLoS One 7:e48737. 10.1371/journal.pone.0048737 23139815 PMC3490853

[6] McArthur AG Waglechner N Nizam F Yan A Azad MA Baylay AJ Bhullar K Canova MJ De Pascale G Ejim L Kalan L King AM Koteva K Morar M Mulvey MR O’Brien JS Pawlowski AC Piddock LJV Spanogiannopoulos P Sutherland AD Tang I Taylor PL Thaker M Wang W Yan M Yu T Wright GD 2013 The comprehensive antibiotic resistance database. Antimicrob. Agents Chemother. 57:3348–3357 23650175 10.1128/AAC.00419-13 PMC3697360

